# Agreement between test procedures for the single-leg hop for distance and the single-leg mini squat as measures of lower extremity function

**DOI:** 10.1186/s13102-018-0104-6

**Published:** 2018-08-22

**Authors:** Eva Ageberg, Anna Cronström

**Affiliations:** Department of Health Sciences, Lund University, Lund, Sweden

**Keywords:** Lower extremity, performance-based measures, hop performance, postural orientation, reproducibility of results

## Abstract

**Background:**

Different test procedures are often used within performance-based measures, causing uncertainty as to whether results can be compared between studies. Thus, the aim of this study was to assess agreement between different test procedures for the single-leg hop for distance (SLHD) and the single-leg mini squat (SLMS), respectively, two commonly used tasks for assessing deficiency in lower extremity muscle function.

**Methods:**

Twenty-three participants (20–42 years) with lower extremity injury performed the SLHD with arms free and with arms behind back, and the Limb Symmetry Index (LSI; injured leg divided by uninjured and multiplied by 100) was calculated. Another group of 28 participants (mean 18–38 years) performed five SLMSs at a pre-defined speed and maximum number of SLMSs during 30 seconds, and were visually observed and scored as either having a knee-over-foot or a knee-medial-to-foot position (KMFP).

**Results:**

No systematic difference between test procedures for the LSI of the SLHD was noted (*p=*0.736), Cohen’s kappa = 0.42. The Bland & Altman plot showed wide limits of agreement between test procedures, with particularly poor agreement for participants with abnormal LSI (<90%). Ten participants were scored as having a KMFP during five SLMSs at a predefined speed, while five had a KMFP during maximum number of SLMSs during 30 seconds (*p=*0.063, Cohen’s kappa = 0.56).

**Conclusions:**

The moderate agreement between the two test procedures for the SLHD and the SLMS, respectively, indicate that results from these different test procedures should not be compared across studies. SLHD with arms behind back, and five SLMSs at a pre-defined speed, respectively, were the most sensitive procedures to detect individuals with poor functional performance.

## Background

Performance-based measures (PBM) are commonly used as screening tools to identify athletes with higher risk of injury [1–3] as well as to assess deficiency in muscle function, and effects of interventions on muscle function, in people with lower extremity injury [4–7]. PBMs have the advantage that they are easy to administer in the clinical setting and in research because they require little equipment and are quickly accomplished. Several, more or less evidence-based PBMs, such as the single leg squat and different jump landing tasks are available in the clinical and sporting settings [8–11]. Two valid [11] and reliable [8, 12] and commonly used PBMs are the single-leg mini squat (SLMS), resembling activity of daily living, for visual assessment of medio-lateral knee position [12, 13], and the single-leg hop for distance (SLHD) [5, 14], resembling more demanding sporting activity. However, different test procedures are often used within each task, causing uncertainty as to whether results can be compared between studies [5, 12–14].

The SLHD is performed either with arms behind back or with arms free during the execution of the hop, and the Limb Symmetry Index (LSI) is typically used to assess differences in hop distance between the injured and uninjured legs [5]. The knee position relative to the foot is assessed and scored during either the performance of five SLMSs at a predefined speed [12] or during the performance of maximum number of SLMSs during 30 seconds [13]. The aims of the current study were to assess agreement between the different test procedures within the tasks for the SLHD and the SLMS, respectively and to determine if any of the within task procedure is more sensitive to detect individuals with worse functional performance than the other.

## Methods

### Participants

As a sample of convenience, two groups of participants were included. Since the LSI between the injured and non-injured legs is most frequently used when evaluating the SLHD, one group with lower extremity injury performed the SLHD (group 1). The SLMS, on the other hand, is commonly used as a screening task for detecting undesirable knee movement patterns in healthy individuals and thus, another group of non-injured individuals performed the SLMS (group 2). Patient demographics were collected prior to the assessment of the functional tasks. The participants were recruited among physical therapy students at the Faculty of Medicine, Lund University, Sweden and screened for eligibility by the assessors (physical therapists). The Advisory Committee for Research Ethics in Health Education at the Faculty of Medicine, Lund University approved the study (VEN 78-10, VEN 91-10) and the participants gave their written informed consent.

#### Group 1

For the assessment of the SLHD, 23 participants (16 women) were included. The inclusion criteria were: i) 18 to 45 years, ii) any injury to the hip, knee or foot causing perceived functional limitations (functional limitations are similar in people with an injury or disease [4, 15]) representing at least monthly awareness of hip/knee/foot problem (subscale quality of life, question 1) in the Hip dysfunction and Osteoarthritis Outcome Score (HOOS), Knee injury and Osteoarthritis Outcome Score (KOOS), or Foot and Ankle Outcome Score (FAOS), as appropriate [16–18] , iii) ability to perform a single-leg hop for distance. The HOOS, KOOS and FAOS are valid and reliable questionnaires, developed to assess patients’ opinions about their hip, knee and foot, respectively, in patients with and without osteoarthritis [18–20]. Volunteers were excluded if they had other injuries or diseases overriding the hip, knee or foot symptoms, or if they, for any reason, were unable to perform the SLHD. Three participants had bilateral lower extremity injury. In those cases, the leg causing most perceived problems was determined as injured. All participants were physically active [21] (Table 1). Their KOOS/HOOS/FAOS scores were comparable to other cohorts with lower extremity injury [16, 18, 22], suggesting that this group was representative for the purpose of this study.

**Table 1:** Characteristics of participants. Participants in cohort 1 were assessed with the single-leg hop test for distance and those in cohort 2 were assessed with the single-limb mini squat.

Characteristic	
*Cohort 1 (n=23)*	
Age (y), mean (SD)	24 (4.9)
Women, (n)	16
BMI (kg/m^2^), mean (SD)	22.2 (2.0)
Injured joint, (n)	
Hip	2
Knee	12
Foot	9
Injured side, right/left (n)	14/9
Grimby physical activity level, median (quartiles)	5 (4–6)
HOOS/KOOS/FAOS subscales	
Pain	87 (11.2)
Symptoms	76 (15.7)
ADL	96 (9.3)
Sport/Rec	72 (19.7)
QOL	64 (18.7)
*Cohort 2 (n=28)*	
Age (y), mean (SD)	23 (4.1)
Women (n)	15
BMI (kg/m^2^), mean SD	22.8 (2.5)
Grimby physical activity level, median (quartiles)	5 (4–6)

*y* years, *SD* standard deviation, *n* number of subjects, *BMI* body mass index. The Grimby physical activity scale ranges from 1-6, least to hardest physical activity. A level of 4 corresponds to moderate exercise 1-2 hours a week, a level of 5 is equal to moderate exercise at least 3 hours a week, and a level of 6 is equal to hard or very hard exercise several times a week. *HOOS* Hip Osteoarthritis Outcome Score, *KOOS* Knee Osteoarthritis Outcome Score, *FAOS* Foot Osteoarthritis Outcome Score, *ADL* activity of Daily Living, *QOL* Quality of Life

#### Group 2

For the assessment of the SLMS, 28 physically active participants (15 women), 18 years or older were included (Table 1). People with any injuries limiting their daily activities and/or their ability to complete the squatting test were excluded.

### Assessment

#### Single-leg hop for distance (SLHD)

**Fig. 1:**
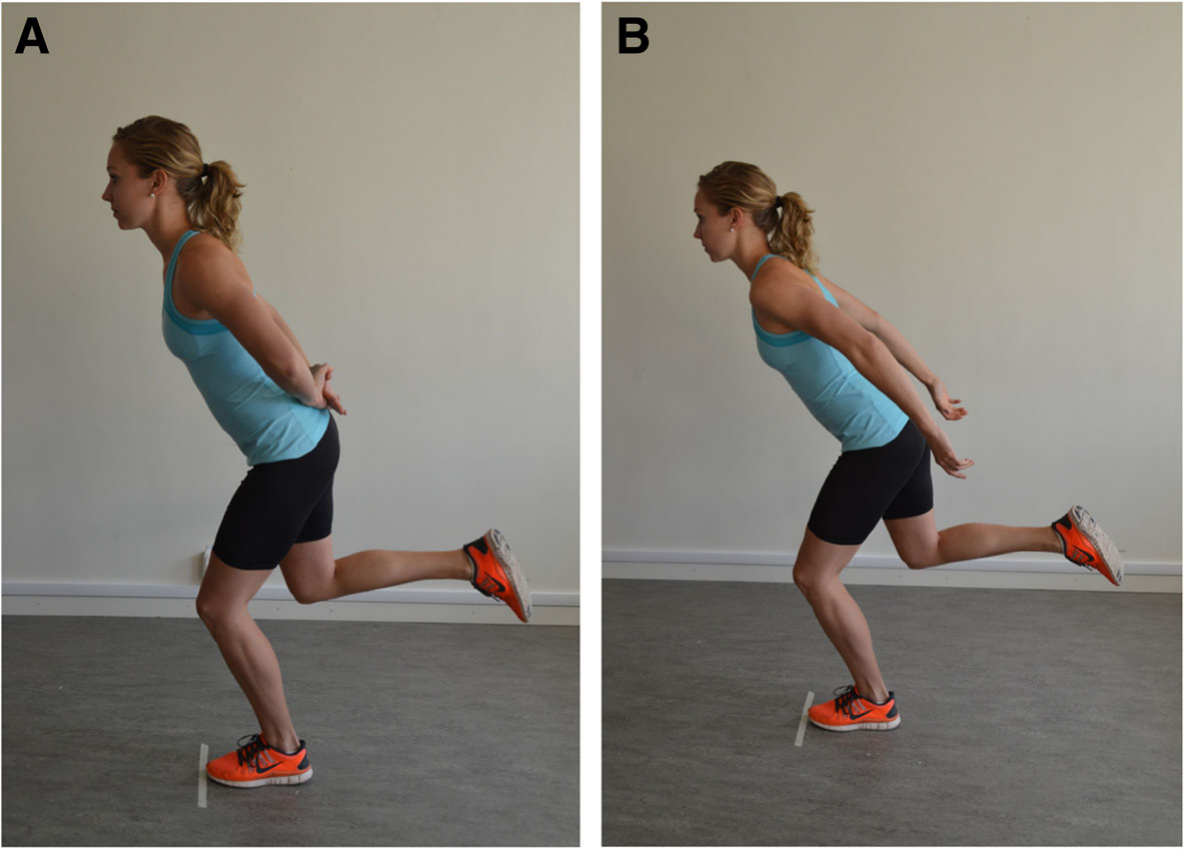
Single-leg hop for distance performed with hands behind back (**a**) and with arms free (**b**).

The participants performed the SLHD with their hands placed behind their back [23], and with the arms free [24] aiming at a more functional execution of the hop, in a randomized [25] order (Fig. 1). The participant stood on the tested leg with the toes behind a marked line, and with the other leg lifted from the floor by flexing the knee. The participants were told to hop as far as possible, taking off and landing on the same foot, maintaining their balance for about 2-3 seconds. The test was performed three times with each leg, alternating the right and left leg. The hop distance (cm) was measured from toe in the starting position to heel in the landing position with a measuring tape by the same examiner. If the participant improved more than 10 cm between the second and third hop, additional hops were performed until an increase of less than 10 cm was measured. One trial hop preceded the measurements. The participants wore their own shoes, e.g., sneakers.

#### Single-limb mini squat (SLMS)

**Fig. 2:**
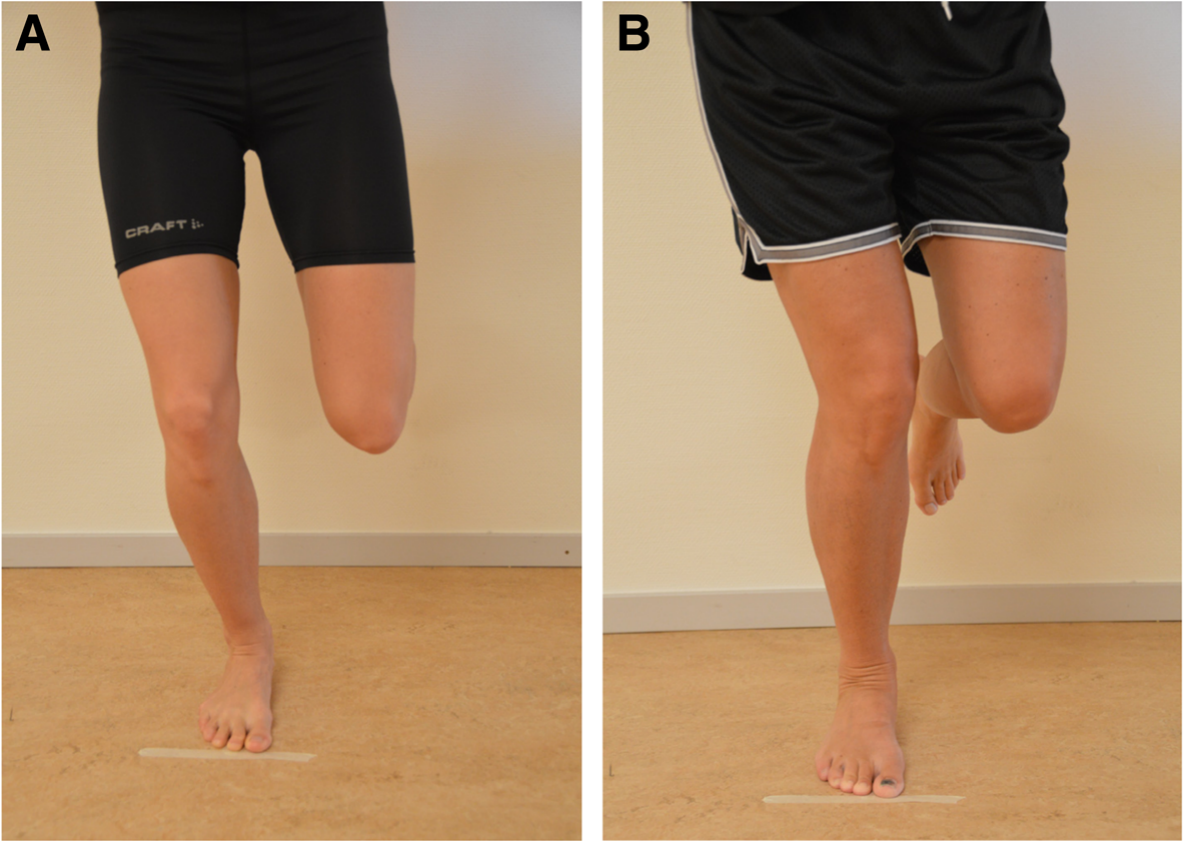
Single-limb mini squat visually observed and scored as knee-over-foot (**a**) or knee-medial-to-foot (**b**).

The participants performed five SLMSs at a pre-defined speed of 20 squats/min (i.e., 3 seconds from starting position to the knee flexion position and back to the starting position) [12] and maximum number of single-limb squats during 30 seconds [13]. The tasks were performed in a randomized order, to avoid any fatiguing effects. The right leg was tested in all participants, and the participants were barefoot during the test [12]. The procedure for the SLMSs was as follows: A “T” was marked with tape on the floor. The patient stood with the long axis of the foot aligned to the stem of the “T”; the second toe placed on the stem. The other leg was kept with the hip in slight flexion and the knee in about 80 degrees of flexion. Finger-tip support for balance was provided by the examiner. The participant was then asked to look down and bend his/her knee, without bending forward from the hip, until he/she no longer could see the line along the toes (corresponding to about 50 degrees of knee flexion), and then return to extension. Three practice trials preceded the SLMS at a pre-defined speed and a 10 second trial preceeded the SLMSs during 30 seconds. The examiner were placed in front of the subject [12, 13] and thhe participant was scored as either having a knee over foot position (KOFP) or a knee medial to foot position (KMFP). A KOFP was scored when the knee was well aligned over or lateral to the 2^nd^ toe in three or more of five trials for the SLMSs at the pre-defined speed, and in the majority of the maximum number of SLMSs during 30 seconds. A KMFP was scored when the knee was placed medial to the 2^nd^ toe (Fig. 2). Two examiners separately assessed and scored the participants’ knee position relative to the foot in real time. After each participant was assessed, the two examiners discussed the scoring of the knee position and a consensus was used in the analysis. The examiners were well trained by an experienced examiner, from pilot testing preceding the present study. The participants were not told that the knee position relative to the foot was assessed during the tests.

### Statistical analysis

For the SLHD, the mean value of the three hops was used in the analysis. Analysis without the 3 participants with bilateral injury did not alter the results; therefore, data is given for all participants. The LSI value, calculated by dividing the result for the injured leg by that of the uninjured leg and multiplying by 100, was used for comparison between the two test procedures (hands behind back vs arms free during the execution of the hop). The mean difference and 95% CI, and “Bland & Altman plots” (scatter plots of the difference between the methods against their mean) with limits of agreement (LOA) [26, 27] were used for assessing agreement between the two test procedures. The “Bland and Altman plot” is the preferred statistical test when assessing agreement between different test procedures and shows systematic differences as well as heteroscedasticity, i.e., larger variability in one end of the measuring scale [26, 27]. In our data for the SLHD, the variability was much larger when the LSI was low. A logarithmic transformation could possibly decrease this relationship [27]. However, logging the data for the Bland & Altman plot did not change the result, and, therefore, the original data is presented. The LSI was also dichotomized into two groups: one group including the participants that had an LSI of 90 or above (normal LSI), and one group that had an LSI below 90 (abnormal LSI) [5]. Mc Nemar’s test was used to test the proportion of normal vs abnormal LSI for the SLHD, and KMFP vs KOFP for the SLMS, between the test procedures. Cohen’s Kappa coefficient was used as a measure of agreement between the procedures. The following thresholds for the Cohens’s Kappa coefficient were used; <0.00 poor agreement, 0.00-0.20 slight agreement, 0.21-0.40 fair agreement, 0.41-0.60 moderate agreement, 0.61-0.80 substantial agreement, 0.81-1.00 almost perfect agreement [28]. Pearsons’ correlation coefficient and Spearmans’ rank correlation coefficient were used as appropriate to assess possible associations between demographics and performance during the SLHD and SLMS, respectively. A level of *p*≤0.05 was chosen to indicate statistical significance.

## Results

There were no associations between demographics and medio-lateral knee position for the two procedures for the SLMS. A lower activity level was associated with a higher LSI for arms behind back (*r=*0.41, *p=*0.049) during the SLHD.

### Single-leg hop for distance

**Fig. 3:**
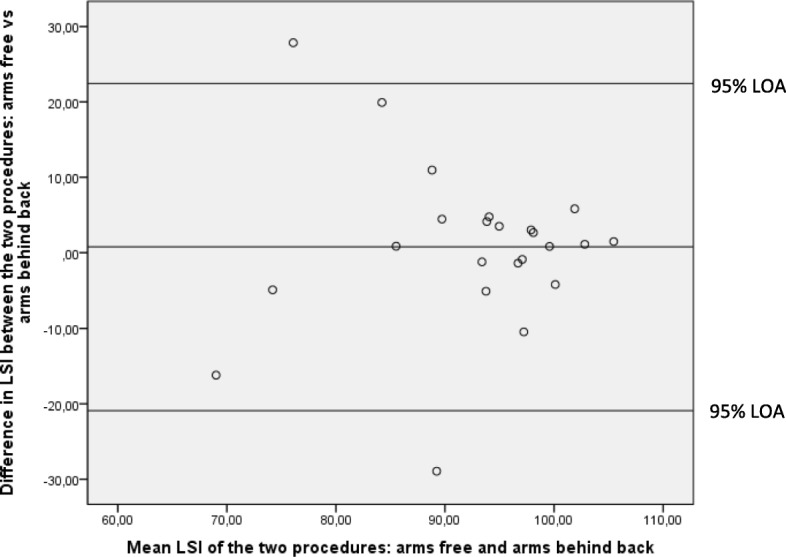
Bland & Altman graph with limits of agreement (LOA) for the Limb Symmetry Index (LSI) of the single-leg hop for distance (SLHD). The differences in LSI between the test procedures with arms free and arms behind back plotted against their mean LSI with the 95% LOA. Mean difference 0.79 (95% LOA -20.91 to 22.44).

The participants hopped a shorter distance with their injured leg than their uninjured leg, with hands behind their back and with arms free (p≤0.004) (Table 2), indicating functional deficiency. There was no systematic difference between the two test procedures for the LSI (Table 2). The Bland & Altman plot revealed a wide LOA between the two procedures with particularly poor agreement when participants had abnormal LSI (Fig. 3). Cohen’s kappa was 0.416 (95%CI 0.010 to 0.822, *p=*0.033). Cross-tabulation showed poor agreement between the procedures for the proportion of participants with abnormal LSI (*p=*0.375) (Table 3).

**Table 2:** Mean (SE) for injured (inj) and uninjured (uninj) legs, mean difference (95% CI) between legs (cm, %), Limb Symmetry Index (LSI, %) for the single-leg hop for distance (SLHD) performed with hands behind back and with arms free (n=23).

	Inj (cm)	Uninj (cm)	Inj vs Uninj (cm)	LSI (%)	Hands behind back vs arms free (%)
	Mean (SE)	Mean (SE)	Mean diff (95% CI)	Mean (SE)	Mean diff (95% CI)
Hands behind back	110.3 (6.3)	119.1 (5.5)	-8.8 (-14.1; -3.7)	91.9 (2.3)	0.79 (-3.99; 5.56)
Arms free	132.8 (7.1)	142.2 (6.0)	-9.4 (-15.0; -3.4)	92.7 (2.2)	

*SE* standard error, *diff* difference

**Table 3:** Cross tabulation of the proportion of normal and abnormal LSI between the two procedures for the single-leg hop for distance (SLHD).

		SLHD, arms free		Total
		Normal LSI	Abnormal LSI	
SLHD, arms behind back	Normal LSI	15	1	16
	Abnormal LSI	4	3	7
Total		19	4	23

*LSI* Limb Symmetry Index

### Single-limb mini squat

Ten participants were scored as having a KMFP during the performance of five SLMSs at a predefined speed of 20 squats per minute, and five participants during the performance of maximum number of SLMSs during 30 seconds (*p=*0.063) (Table 4). Cohen’s kappa was 0.56 (95% CI 0.251 to 0.875, *p=*0.001), reflecting the agreement between the two test procedures.

**Table 4:** Cross tabulation of the proportion assessed with a KOPF and a KMFP, respectively during the two test procedures for the single-limb mini squat (SLMS).

		Maximum number of SLMS for 30 seconds		Total
		KOFP	KMFP	
5 SLMS	KOFP	18	0	18
	KMFP	5	5	10
Total		23	5	28

*KOPF* knee Over Foot Position, *KMFP* Knee Medial to foot postion

## Discussion

Nearly twice as many participants had abnormal LSI with arms behind back (30%) compared to the procedure with arms free (17%) during the SLHD, indicating that the hop test performed with arms behind back is better at detecting individuals with functional limitations than arms free. The Bland and Altman plot showed that the agreement between the procedures seems to be good when the participants had an LSI close to normal, but worse when the participants had lower LSI. This indicates that the result from studies using these two different procedures (arms behind back vs arms free) should not be compared.

A well-known limitation of the LSI is that an injury may affect also the uninjured leg. That is, a small difference between the injured and uninjured legs may be due to impaired function of both legs, causing an overestimation of the LSI and, consequently, function of the knee [29]. This misinterpretation may lead to a return to sport too early after injury and, thus an increased risk of re-injury [29]. Despite this limitation, results from several studies suggest that the LSI is a useful measure. Longitudinal studies report that a low LSI predicts poor long-term outcomes, such as worse perceived function [30, 31], less likelihood of returning to sports [32], and OA development [33]. The LSI is also a useful variable in order to minimize the influence of multiple testers and different testing devices [34], and it is widely used as a return to sport criterion [5]. However, when using the LSI as a measure of function after injury, the result from this study indicates that performing the tasks with arms behind back may be more demanding and thus more sensitive in detecting muscle function deficiency compared to arms free in this group of patients. Also, since this study included participants with different injuries, i.e., hip, knee and foot injuries, this result may be generalizable to patients with a wide range of musculoskeletal complaints. Further studies may reveal if arms behind back is the most appropriate SLHD protocol for predicting other outcomes as well, such as return to sport and perceived function in patients with injury to the lower extremity.

Twice as many participants were scored as having a KMFP during the performance of five SLMSs at a predefined speed of 20 squats per minute (40%), than during the performance of maximum number during 30 seconds (20%). The difference did not reach statistical significance (*p=*0.063). However, it indicates that five SLMSs at a slow speed may be better at detecting individuals with functional limitations, and the difference was considered clinically relevant for the purpose of this study. Also, the kappa value of 0.56 suggests only a moderate agreement between the two test procedures implying that these procedures should not be used interchangeably. Taken together, this suggests that results from studies using these different test procedures should not be compared.

A medially placed knee in relation to the foot is more common in women compared with men [35] and is, thus, suggested to be associated with the higher knee injury risk observed in females [36]. This altered movement pattern is also reported to be related to worse perceived knee function [37–39], worse knee confidence [40] and worse hop performance [37] in patients with lower extremity injury, implying that this is a valuable measure of functional performance. The observed moderate correlation between postural orientation errors (measurement of joints in relation to each other) and hop tests [37], indicate that these measures encompass different aspects of performance and that both can be used to obtain a complete picture of the patient’s function. It may be valuable in future studies to record the performance of the SLMS during 30 seconds on video, so that the knee position in relation to the foot can be viewed repeatedly and/or in slow motion [41]. This would determine whether the difference in our study is due to the difficulty of assessing the knee position relative to the foot in real time or if 5 slow squats is a more sensitive test for detecting a KMFP during a SLMS.

This study has some limitations. Lower Grimby activity level was associated with higher LSI during the arms behind back procedure for the SLHD (*r=*0.41, *p=*0.049). However, the correlation coefficient between the activity level and the SLHD with the arms free was quite similar (*r=*0.35, *p=*0.104) but did not reach statistical significance. Since the activity level seems to affect the LSI in a similar manner for both procedures, we do not believe that this have had an effect on the results in this study. Also, since this is a cross-sectional study, we cannot draw any conclusions regarding causal relationship between activity level and LSI during the hop tests. Another limitation is the small sample size. A sample of at least 50 individuals is recommended in agreement studies [42]. Even though the result from this study seems to be sufficiently evident, it is possible that a larger sample may have altered the result.

## Conclusions

Comparisons should not be made between groups of participants with lower extremity injury for the LSI of the SLHD when the different test procedures arms free or behind back during the performance of the hop have been used. Neither should results for medio-lateral knee position during the performance of the two SLMS protocols be compared. SLHD with arms behind back and 5 SLMS at a pre-defined speed may be preferred to identify individuals with worse function as assessed by hop distance and medio-lateral knee position, respectively.

## Abbreviations

FAOS: Foot and ankle outcome score
HOOS: Hip dysfunction and osteoarthritis outcome score
KMFP: Knee medial to foot position
KOFP: Knee over foot position
KOOS: Knee injury and osteoarthritis outcome score
LOA: Limits of agreement
LSI: Limb symmetri index
PBM: Performance based measures
SLHD: Single leg hop for distance
SLSM: Single leg mini squat
